# COUPLE COMMUNICATION AFTER RETIREMENT AND WELL-BEING IN ARAB SOCIETY: AN APIM ANALYSIS

**DOI:** 10.1093/geroni/igae098.2515

**Published:** 2024-12-31

**Authors:** Reem Nashef-Hamuda

**Affiliations:** Haifa University, Haifa, HaZafon, Israel

## Abstract

This study examined the influence of perceived marital responsiveness on life satisfaction and depression among 106 retired married couples from the Arab society in Israel (mean male age = 70.3, female age = 65.1) with an average of 43.3 years of marriage and 3-5 children. Utilizing the Actor-Partner Interdependence Model (APIM), findings highlighted a positive impact of individual (actor) responsiveness on Satisfaction with Life Scale (SWLS) scores and a negative impact on depression levels. Furthermore, partner responsiveness significantly affected both life satisfaction and depression, underscoring the importance of mutual perceptiveness in marital dynamics. The analysis revealed no significant relationship between marital control and either SWLS or depression, nor were there significant gender differences in the effects, indicating that the benefits of perceived responsiveness are consistent across genders. These results emphasize that life satisfaction and depression in retirement are influenced by both one’s own and one’s partner’s responsiveness, reinforcing the value of mutual support in marital relationships.

